# Advancements in nanomedicines for cancer therapy: targeting molecular signaling pathways, activating immune cells, and using photothermal immunomodulation

**DOI:** 10.3389/fbioe.2026.1775442

**Published:** 2026-04-10

**Authors:** Vishal Kumar Deb, Abhishek Mukherjee, Surajit Pathak, Sujay Paul, Asim K. Duttaroy, Suman Adhikari

**Affiliations:** 1 School of Health Sciences and Technology, UPES, Dehradun, Uttarakhand, India; 2 Ambinova Technologies Pvt Ltd., New Delhi, India; 3 Faculty of Allied Health Sciences, Chettinad Academy of Research and Education (CARE), Chettinad Hospital and Research Institute (CHRI), Chennai, India; 4 Tecnologico de Monterrey, School of Engineering and Sciences, Querétaro, Mexico; 5 Department of Nutrition, Institute of Medical Sciences, Faculty of Medicine, University of Oslo, Oslo, Norway; 6 Department of Chemistry, Govt. Degree College, Dharmanagar, India

**Keywords:** cell signaling-target, clinical transition, immune cell activation, nanomedicine, photothermal therapy, two-domensional nanomedicine

## Abstract

Nanoparticles are known for their unique physicochemical characteristics and large surface area. When these nanoparticles are functionalized with bioactive molecules or proteins, they enable highly efficient and targeted applications in cancer therapy. Early applications of nanotechnology in cancer treatment have centered on the properties of various nanomaterials, including organic, inorganic, and biological nanoparticles. For instance, stability, large-scale synthesis, and potential toxicity are crucial determinants of the use of nanotechnology in cancer treatment. For specific intervention, nanotechnology has focused on targeting dysregulated cancer signaling pathways. Concurrently, reprogramming tumor-associated macrophages, activating T and natural killer cells, and modulating cytokine profiles have become key components of nano-immunotherapy in combating tumor growth. Biomimetic nanotechnology, particularly nanoparticles wrapped in cancer cell membranes and photothermal immunotherapy based on two-dimensional (2D) nanomaterials, has shown potential as a new approach for cancer immunotherapy. Nevertheless, specific gaps in combination therapy, such as dose optimization and safety considerations, remain to be addressed in translational research. This review highlights mechanistic insights into nanoparticles that modulate cellular signaling pathways and activate immune cells, emphasizing 2D material-based nano-immune delivery systems as promising next-generation cancer treatments, with particular emphasis on photothermal therapy that could advance from preclinical to clinical settings.

## Graphical Abstract

**Figure:**
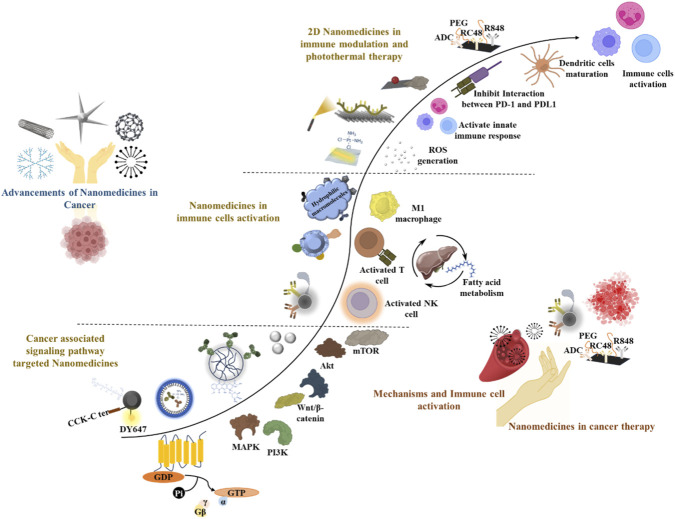

## Highlights

- Nanodrug carriers facilitate photothermal therapy.
- Nanomedicines target dysregulated cellular pathways.
- Nanodrugs activate immune cells and use a biomimetic approach to treat cancer.
- Nanomaterials have translational potential as photo-immunotherapeutic agents.

## 1 Introduction

Cancer is a major global health concern, with millions affected by its devastating impact; it is the second largest cause of mortality globally (Bray et al., 2024; Siegel et al., 2025). Despite significant advancements in understanding cancer biology and the development of various treatment modalities, the management of this complex disease remains challenging (Debela et al., 2021; Liu et al., 2024). Conventional cancer therapy, including chemotherapy, surgery, and radiation therapy, has substantially contributed to patient survival. However, these approaches often suffer from limitations, including systemic toxicity, nonspecific targeting of normal cells, and drug resistance, necessitating the exploration of alternative strategies (Nath et al., 2022; 2024; Das et al., 2023b; Adhikari et al., 2024; Garg et al., 2024). Nanotechnology has evolved as a promising field with the potential to revolutionize cancer treatment (Bravo-Vázquez et al., 2023; Ochoa-Sanchez et al., 2024; Sabir et al., 2025; Uriostegui-Pena et al., 2025). The use of nanoparticles (NPs) in cancer therapy capitalizes on their distinctive features, including small size, tunable surface properties, and a large surface area-to-volume ratio (Lan et al., 2023). These attributes enable NPs to interact with biomolecules at the molecular level, delivering a stage for specific, targeted drug delivery. Furthermore, NPs provide precise delivery of diverse cargoes such as chemotherapeutic drugs, peptides, nucleic acids, and antibodies, resulting in controlled release and enhanced bioavailability (Singh et al., 2021; Das et al., 2023a; 2023b). NPs, with their inimitable physicochemical features and tailored characteristics, offer unprecedented opportunities for combating cancer. A key feature of NPs is their ability to accumulate selectively within tumor tissues (Gavas et al., 2021). This phenomenon, known as the enhanced permeability and retention (EPR) effect, depends on the leaky vasculature and poor lymphatic drainage commonly found in solid tumors. NPs can exploit these characteristics to preferentially accumulate at tumors, resulting in high drug concentrations and improved therapeutic efficacy. By leveraging the exceptional properties of nanomaterials, advanced approaches are being adopted to improve drug delivery, enhance therapeutic efficacy, and overcome challenges in conventional cancer treatment approaches.

Organic NPs, including liposomes and polymeric NPs, have earned significant consideration in cancer research (Palazzolo et al., 2018; Singh et al., 2021). Liposomes are phospholipid bilayers that can capture both hydrophobic and hydrophilic drugs. Liposomes’ biocompatibility and versatility make them a good choice for drug delivery (Allahou et al., 2021). Polymeric NPs offer various formulation options, thereby enabling controlled release of therapeutics and simultaneous targeting of multiple factors (Kamaly et al., 2016). Inorganic NPs, such as metal-based NPs (e.g., Au and Ag) and quantum dots (QDs), have exceptional physicochemical properties that can be explored for various applications in cancer therapy (Liu et al., 2021; Mazumdar et al., 2025). Metal-based NPs exhibit optical properties, such as surface plasmon resonance, that enable their use in photothermal therapy, imaging, and targeted drug delivery (Mariano et al., 2024). Recently, QDs, semiconductor nanocrystals with size-tunable fluorescent properties, have emerged as powerful tools for cancer diagnosis and imaging (Mukherjee et al., 2016). Biological-based NPs, such as exosomes and virus-like particles (VLPs), have earned substantial attention as therapeutic modalities (Kim et al., 2023; Rahmat et al., 2024). Exosomes are nanosized extracellular vesicles secreted by many cells, including tumor cells. They can be employed as natural transporters for drug delivery and for diagnostic and therapeutic purposes. VLPs, which mimic the structure and properties of viral particles, have shown promise in cancer immunotherapy as antigen-presenting platforms or carriers of immunostimulatory molecules (Shahrivarkevishahi et al., 2022).

Clinical translation of nanoformulated therapeutics is a critical milestone towards realizing the potential of NPs in cancer treatment (Mundekkad and Cho, 2022; Zhang et al., 2023). Several nanoformulations have already advanced to clinical trials, demonstrating promising results in improving drug distribution, enhancing effectiveness, and reducing side effects (Caster et al., 2017; Shukla et al., 2021; Asadi et al., 2025). By evaluating the safety and efficiency of nanoformulated therapies in humans, valuable insights can be gained to pave the way for their integration into standard cancer-treatment protocols. NPs have demonstrated significant potential in cancer immunotherapy, a rapidly growing research arena that harnesses the human immune system to fight cancer (Lahiri et al., 2023; Fallatah et al., 2025). NPs play a substantial role in enhancing the efficiency of immunotherapeutic approaches by performing as carriers for immunomodulatory agents, improving antigen presentation, and strengthening immune-cell targeting. Additionally, NPs can be designed to conquer immunosuppressive mechanisms in the tumor microenvironment (TME), thereby enabling highly effective immune responses against cancer cells (Lu et al., 2024).

While the use of NPs in cancer therapy is promising, some issues remain to be addressed (Hasan et al., 2025). The synthesis and characterization of NPs and their scaled-up production need to be optimized to ensure reproducibility and regulatory compliance. Safety considerations, including potential toxicity and long-term effects, must be systematically assessed to minimize the risk associated with NP-based therapies. Cancer therapy is on the cusp of a significant revolution driven by nanotechnology (Wen et al., 2025). Ongoing clinical trials and research efforts are crucial for translating NP-based therapies into clinical practice, eventually improving patient outcomes and transforming the landscape of cancer therapy (Mangla et al., 2025). This review emphasized the current advances in nanomaterials for targeting cancer-associated cell signaling pathways and transitioning toward immune cell activation to eradicate cancer cells, as well as the most recent updates in photothermal immunotherapy. It also discussed the molecular mechanisms and therapeutic interventions translating into clinical prospects.

## 2 Cancer signaling pathways targeted by NPs and the mechanistic approach

### 2.1 NPs targeting the G-protein-coupled receptor (GPCR) pathway

GPCR is one of the largest seven-transmembrane cell-surface receptors, and it is the most prominent family of surface receptors in humans (Bar-Shavit et al., 2016). GDP is converted to GTP during GPCR activation following ligand binding, forming a GTP-bound Gα monomer and Gβγ dimer. Interestingly, GPCR-related signaling pathways become dysregulated and are highly likely to induce cancer as a result of *GPCR* mutations (Liu et al., 2016). In this regard, synthetic therapies based on nanomaterials are attracting attention for targeting GPCRs in cancer therapy. For example, AuNP conjugated with non-peptide GPCR agonists has demonstrated potential as an anti-cancer drug (Jayasekara et al., 2013). Additionally, their characteristics are comparable to those of the monomeric form of activated GPCR, which helps selectively identify the receptor and attenuate cAMP generation.

**FIGURE 1:**
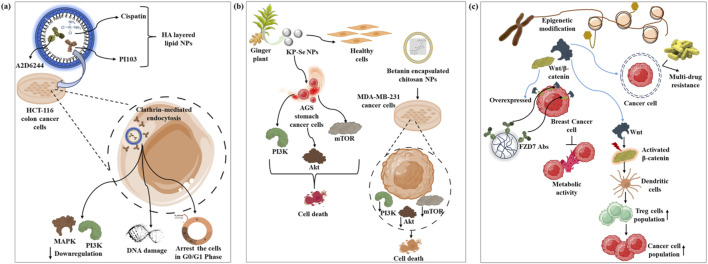
**(a)** Hyaluronic acid-modified lipid-based chimeric NPs conjugated with AZD6244 (MAPK inhibitor), cisplatin, and PI103 (PI3K inhibitor), inhibiting MAPK and PI3K proteins, inducing DNA damage, and arresting the cells in G0/G1 stage in colon cancer cells; **(b)** *Kaempferia parvifora* extract and selenium salts based KP–SeNP targeting AGS cancer cells, decreasing the expression of phosphorylated PI3K/Akt/mTOR; **(c)** Doxorubicin encapsulated PLGA NPs conjugated with an anti-FZD7 antibody suppress the overexpressed triple-negative breast cancer receptors.

Sanchez et al. have synthesized iron oxide NPs (IONPs) to target the GPCR cholecystokinin-2 receptor (CCK2R). Thyroid, lung, and gastrointestinal cancer cells express CCK2R. Gastrin and CCK are crucial in activating this receptor. A synthetic gastrin, MG, generated using the C-terminus of CCK and conjugated to IONP has been developed to target CCK2R. Cancer cells expressing CCK2R have undergone receptor-mediated endocytosis. Crucially, IONPs have been found to induce cell death *via* lysosomal leakage when a magnetic field was introduced as an external stimulus (Figure 1a) (Sanchez et al., 2014; Ma et al., 2018).

C-X-C motif chemokine ligand (CXCL) 12/C-X-C motif chemokine receptor (CXCR) four antagonists and nanoscale drug delivery systems have been used to treat individuals with various myeloma and non-Hodgkin lymphoma. Plerixafor (AMD3100), a small non-peptide molecule, has been incorporated in a nano-drug delivery system that interacts with CXCR4, which is overexpressed in non-Hodgkin’s lymphoma and multiple myeloma, to prevent the binding of CXCL12 (Teicher and Fricker, 2010) and attenuate the downstream signaling cascades (Zhao R. et al., 2022). GPR-87, which is overexpressed in pancreatic cancer, plays a vital role in tumor growth. To suppress its expression, NPs have been used as vehicles to deliver nucleosides, achieving a relatively high degree of internalization and stability in the blood circulation. In this study, small-interfering RNA (siRNA) encapsulated within poly (lactide-co-glycolide) (PLGA) has been found to silence GPR-87 expression in GPCR87-transfected HEK 293T cells (Table 1) (Ceylan et al., 2020). Abnormal expression of CXCR4 has been documented in several malignancies (Ansari et al., 2023). Recently, a tailored drug-delivery method has been developed to target the CXCR4 ligand DV1 peptide for killing cancer cells that highly express CXCR4 (Table 1). Avidin-PLGA NPs have been used to conjugate DV1 peptide ligands to drug-loaded nanoparticles, which have been evaluated for possible clinical use in cancer cells expressing CXCR4 (Ansari et al., 2023).

**TABLE 1:** Different nanoparticles in cancer-associated cell signaling pathways target.

Name of nano-formulation	Types of cancer	Nanomaterials characterization techniques used	Targeted signaling pathway protein	Effective dose concentration/IC_50_ concentration	*In-vitro*/*In-vivo* model	Effects	References
siR-PLGA-V/S	Pancreatic cancer	DLS, Zeta Potential (ZP), TEM	GPCR	62 ng/mL and 31 ng/mL	1.1B4 cancer cell	• Silence GPR-87 gene expression	Ceylan et al. (2020)
avidin-poly (lactic-co-glycolic acid) (PLGA) nanoparticle	Glioblastoma	DLS and TEM	GPCR	NR	U87MG cells	• Specificity observed in DV1 peptide-tagged avidin-PLGA nanoparticles towards CXCR4 receptors • Potential therapeutic effect based on the CXCR4 targeting receptor expressed in cancer cells	Ansari et al. (2023)
Hyaluronic Acid-Coated Chimeric Nanoparticles (Consisting of AZD6244 (MEK inhibitor) and PI103 (dual AktmTOR inhibitor)	Colon cancer	DLS, Zeta Potential (ZP), FE-SEM, AFM, and TEM	MAPK-PI3K	IC_50_ = 0.91 ± 0.09 μM	HCT-116 cells	• Attenuated MAPK-PI3K signaling • Induced DNA damage • Cancer cells arrested in G0/G1 phase and triggered apoptosis	Palvai et al. (2017)
Baicalin-loaded folic acid-modified albumin nanoparticles (FA-BSANPs/BA)	Breast cancer	DLS, Zeta Potential (ZP), FTIR and TEM	MAPK and Akt/mTOR	IC_50_ = 39.3 μM	MCF-7 cells/BALB/c female nude mice	• Arrested the cells in S phase • Induced autophagy Triggered apoptosis *via* ROS-facilitated • p38 MAPK and Akt/mTOR signaling pathway	Liu et al. (2022a)
*amygdalin*-folic acid nanoparticles (Amy-F)	Breast cancer	XRD, FTIR, EDX, SEM, and HR-TEM	MAPK/P38	IC_50_ = 79.8 μg/mL (MCF-7) IC_50_ = 94.9 μg/mL (MDA-MB-231)	MCF-7 and MDA-MB-231 cells	• Encouraged cell cycle arrest in G1 and sub-G1 stages, and enhanced apoptosis • Downregulated MAPK/P38, Fe, NO • Upregulated the ROS level • Improved radiotherapy (RT)	Askar et al. (2023)
KP-SeNP	Gastric cancer	XRD, EDX FTIR, DLS, and TEM	PI3K/Akt/mTOR	200 μg/mL (in cell line) 10 mg/kg (in mice)	AGS cells/Male nude mice	• Upregulated the apoptotic signaling markers including Bcl-2, BAX, and caspase 3 • Increased autophagic flux-marker protein, and LC3B-II • Decreased phosphorylation of PI3K/Akt/mTOR pathway markers	Wang et al. (2022)
Triphenylphosphonium conjugated gold nanotriangles and 5-ALA	Breast cancer	DLS, XRD, TEM, HR-TEM, and EDX	PI3K/Akt	IC_50_ = 0.67 ± 0.89 μg/mL (MCF-7) IC_50_ = 0.58 ± 0.23 μg/mL (MDA-MB-231)	MCF-7 and MDAMB-231 cells	• Improved cellular toxicity • Impairs cell survival PI3K/AKT signaling pathway • Caused mitochondrial-dependent apoptosis	Vinita et al. (2023)
Betanin-encapsulated biopolymeric nanoparticles	Breast cancer	DLS and SEM	PI3K/Akt/mTOR	IC_50_ = 3.974 μg/mL	MDA-MB-231 cells	• Attenuated the expression levels of PI3K, Akt, and mTOR proteins • Prevent cell proliferation • Leads to cell cycle arrest	Rehman et al. (2023)
DOX encapsulated poly (lactic-co-glycolic acid) (PLGA) nanoparticles (NPs) coated with antibodies against Frizzled7 (FZD7)	Breast cancer	DLS, Zeta Potential (ZP), and TEM	Wnt/β-catenin	NR	MDA-MB-231 cells	• Triggered apoptosis and DNA destruction through H2A.X phosphorylation • Superior inhibition of metabolic activity compared to free DOX treatment • Substantial levels of Wnt pathway inhibition • Enhanced in β-catenin phosphorylation, indicative of β-catenin destruction	Hoover et al. (2024)
XAV939 (XAV-Np)	Melanoma	DLS, and HR-TEM	Wnt/β-catenin	NR	B16F10 cells/C57BL/6 mice	• Promoted β-catenin degradation • XAV-Np treatment in macrophages conditioned with melanoma cells significantly upregulated • CD80 and CD86 expression • Augmented IL-6 and TNF-α production and impaired IL-10 production • Increased CD8^+^ T cell proliferation	Pundkar et al. (2023)
Chitosan lactate (CL) based rituximab (RTX) and anti-Nrf2 siRNA conjugated nanoparticles	Chronic lymphocytic leukemia	FTIR, Zeta Potential (ZP), and TEM	Nrf2	IC_50_ (89.34 vs. 145.2 μM)	Patient-derived PBMCs and BMMCs	• Induce cell apoptosis and prevent cell proliferation	Khodakarami et al. (2023)
Nrf2-Targeting siRNA-Loaded in chitosan-shelled nanobubble	Melanoma	DLS, Zeta Potential (ZP), and TEM	Nrf2	0.08-μM	M14 melanoma cells	• Downregulated the target gene that is responsible for the melanoma resistance development	Argenziano et al. (2022)
Metal element gadolinium, AGuIX nanoparticles	Breast cancer	NR	Nrf2-GPX4	0.5 mM	MDA-MB-231 and MDA-MB-468 cells/Female nude mice	• Enhanced DNA disruption by compromising the homologous recombination repair pathway • Induced apoptosis with very low ability • Nanoparticles may control the anti-ferroptosis through NRF2-GSH-GPX4 signaling pathway attenuation	Sun et al. (2022)

### 2.2 NPs targeting the mitogen-activated protein kinase (MAPK) pathway

Disruption of the phosphatidylinositol 3-kinase (PI3K) and MAPK pathways is associated with the development of cancer, such as colon cancer. Targeted delivery using hyaluronic acid-layered lipid-based chimeric NPs coupled with MAPK inhibitor (AZD6244), cisplatin, and PI3K inhibitor (PI103) can be used to treat colon cancer (Table 1). These NPs are rapidly internalized by colon cancer cells *via* CD44 and clathrin-facilitated endocytosis, thereby reducing the expression of MAPK-PI3K signaling proteins and DNA damage. These NPs also cause G0/G1 stage cell cycle arrest and early and prolonged cell death of colon cancer cell. Therefore, hyaluronic acid-coated NPs can be therapeutically used to treat colon cancer (Palvai et al., 2017).

Breast cancer is the most common cancer among females and causes high mortality. Baicalin may potentially slow the growth of breast cancer cells. Albumin NPs conjugated to baicalin-loaded folic acid prevent proliferation of breast cancer cells in the S phase and trigger the release of proapoptotic and autophagic proteins (Table 1). Interestingly, this nanoformulation impairs the expression of signaling proteins that are phosphorylated to promote breast cancer cell proliferation, including phosphorylated Akt and mechanistic target of rapamycin (mTOR) (Liu F. et al., 2022). Amygdalin–folic acid NPs (Table 1) increase the expression of apoptosis-inducing proteins and tumor growth regulators in MDA-MB-231 and MCF-7 breast cancer cells. These NPs hinder the progress of breast cancer cells, with cell cycle arrest at G1 and sub-G1 stages, thereby enhancing the effectiveness of radiation therapy. These NPs may downregulate MAPK/P38 signaling protein expression, as indicated by a thorough molecular analysis (Askar et al., 2023).

### 2.3 NPs targeting the PI3K pathway

PI3K is a crucial determinant of cellular fate. Tumor growth and other biological processes, such as EGFR-activating mutations, are influenced by the PI3K/Akt/mTOR pathway (Miricescu et al., 2020; Adhikari et al., 2025). Akt/mTOR is active in approximately 67% individuals with *EGFR* mutations (Sanaei et al., 2022).

Recent studies are targeting the PI3K/Akt/mTOR pathway for the treatment of gastric cancer. *Kaempferia parvifora* extract and selenium salts (KP–SeNP) have been employed to selectively target AGS stomach cancer cells without affecting healthy cells. The formulation decreased the expression of phosphorylated PI3K/Akt/mTOR (Figure 1b) and increased the level of microtubule-related protein 1 light chain 3 Beta, lipidated form, a marker of autophagic flux. Additionally, KP–SeNPs have demonstrated vigorous anti-cancer activity and biological safety in a xenograft animal model of AGS cancer cells (Wang et al., 2022).

AuNPs have been employed to treat breast cancer cells by photodynamic therapy. Gold nanotriangles (AuNTs) conjugated with triphenylphosphonium and 5-aminolevulinic acid (Table 1) have shown enhanced toxicity to breast cancer cells during photodynamic therapy by promoting mitochondria-dependent apoptosis and reducing PI3K/AKT signaling (Vinita et al., 2023). Similarly, chitosan NPs encasing betanin (CSNP–BET) (Table 1) have been used to kill MDA-MB-231 breast cancer cells. Additionally, MDA-MB-231 cells treated with CSNP–BET have shown a substantial decrease in the expression of PI3K, mTOR, and Akt (Rehman et al., 2023).

### 2.4 NPs targeting the Wnt pathway

Along with the PI3K pathway, the Wnt/β-catenin pathway is crucial for studying anticancer drug resistance. The utilization of β-catenin inhibitors renders patients with triple-negative breast cancer and carrying mutant PIK3CA more sensitive to PI3K inhibitors (Lehmann et al., 2014; Solzak et al., 2017). Additionally, the Wnt pathway and epigenetic alteration are related to multidrug resistance. Chemotherapy combined with targeted therapy for the Wnt/β-catenin signaling pathway produces synergistic effects and decreases Wnt signaling, thereby overwhelming drug resistance in tumor cells (Banerjee and Devi Rajeswari, 2023; Debnath et al., 2024).

Doxorubicin is frequently used as a chemotherapeutic agent for triple-negative breast cancer (TNBC); however, it causes toxicity owing to off-target delivery (Bisht et al., 2025). To address this issue, doxorubicin has been encapsulated in PLGA NPs conjugated to an anti-FZD7 antibody that binds to overexpressed FZD7 receptors in triple-negative breast cancer (Table 1). These overexpressed receptors activate the Wnt signaling pathway in cancer (Figure 1c). The nanoformulation reduced cancer cell proliferation by inhibiting metabolic activity and the Wnt pathway (Hoover et al., 2024). Tumor-targeting metal-organic NPs are manufactured using a dynamic combinatorial chemical method for immunological sensitization-based cancer therapy. This manufacturing method uses a metal-organic coordination polymer, carnosic acid (CA), and a Wnt inhibitor. These NPs have demonstrated a potential tumor-suppressing effect by attenuating Wnt expression *in vitro* and *in vivo* (Liu T. et al., 2022).

Instigation of the Wnt signaling pathway triggers β-catenin signaling in the TME, increasing the number of antigen-presenting cells that mediate anti-cancer activity. In contrast, the activated Wnt/β-catenin pathway in dendritic cells enhances tumor progression by triggering regulatory T cell responses. Tumor-associated macrophages (TAMs) might function as antigen-presenting cells to control anti-tumor immunity, and the role of activated β-catenin in TAMs in the TME remains unclear (Pundkar et al., 2023). To examine the influence of β-catenin suppression on macrophage immunogenicity, melanoma cells co-cultured with melanoma cell supernatants were treated with a tankyrase inhibitor-containing NP formulation of XAV939 (Table 1), which promoted β-catenin degradation. Consequently, XAV-Np treatment of macrophages augmented the expression of CD80 and CD86 while attenuating that of programmed death-ligand 1 (PD-L1) and CD206. Additionally, these NPs increased tumor necrosis factor alpha (TNF-α) and interleukin (IL)-6 expression while decreasing IL-10 expression (Pundkar et al., 2023).

### 2.5 NPs targeting the nuclear factor erythroid 2-related factor 2 (Nrf2) pathway

Inflammation is a pathogenic process in tumor biology, which can occasionally lead to persistent and harmful effects (Dudley, 2012). Nrf2 and nuclear factor kappa B (NF-κB) are two key transcription factors involved in oxidative stress and inflammation (Kaulmann and Bohn, 2014). Additionally, these two proteins work together to control the cellular redox state. In particular, NF-κB stimulates the production of several inflammatory molecules, for example, cyclooxygenase 2 and inducible nitric oxide synthase (Karin et al., 2004). Nrf2 helps reduce reactive oxygen species (ROS) levels and prevent NF-κB activation (Ganesh Yerra et al., 2013; Saha et al., 2024).

Nrf2 plays an intriguing role as a cryoprotector by promoting the expression of genes that may also contribute to the development of melanoma and other malignancies (Moloney and Cotter, 2018; Friedmann Angeli and Meierjohann, 2021). During low oxidative stress, Nrf2 binds to cytosolic Kelch-like ECH-associated protein 1 (Keap1), which increases proteasomal degradation of Nrf2. Several antioxidant enzymes, including γ-glutamate-cysteine ligase, glutathione-S-transferase, and heme oxygenase-1, are expressed during high oxidative stress, and Nrf2 detaches from Keap1 and translocates to the nucleus (Rojo de la Vega et al., 2018). Initially, Nrf2 was believed to protect cells against oxidative stress, thereby preventing malignant transformation. However, elevated Nrf2 levels also promote angiogenesis, cell migration, and resistance to chemotherapy and radiotherapy (LAU et al., 2008; Zhou et al., 2012; Furfaro et al., 2016; Gao et al., 2018). Therefore, siRNA-mediated suppression of Nrf2 may help treat cancer, particularly melanoma, by overcoming Nrf2-mediated chemoresistance. In this regard, chitosan-shelled nanobubbles have been successfully used to deliver siRNA to human melanoma cells M14 (cisplatin-resistant melanoma) for suppressing Nrf2 expression using ultrasound (Table 1) (Argenziano et al., 2022).

Radiation-based cancer treatment is not effective owing to the emergence of resistance. Therefore, current research focuses on developing radiosensitizers to enhance tumor radiosensitivity and mitigate radiation-induced damage to normal cells. Gadolinium metal-based AGuIX NPs have been designed to increase the radiosensitivity of malignancies; however, these NPs can disrupt DNA by interfering with the homologous recombination pathway and initiating apoptosis (Table 1). Conversely, by hindering the NRF2-GSH-GPX4 signaling path in tumor cells, these NPs may exert anti-ferroptotic effects (Sun et al., 2022). A nanodelivery method, NP-Nrf2_siRNA-CP, was developed (Table 1) using chitosan lactate anti-Nrf2 siRNAs, rituximab, and cyclophosphamide. By lowering anti-apoptotic gene expression and increasing proapoptotic gene expression in chronic lymphoblastic leukemia cells, these NPs promoted apoptosis (Khodakarami et al., 2023).

## 3 Nanoparticles as an immunotherapeutic agent for cancer treatment

### 3.1 Nanomaterials in boosting cancer immunotherapy

Modified physicochemical characteristics of NPs are crucial for enhancing therapeutic effects in cancer treatment. Significant advances have been made in cancer treatment by blocking immunological checkpoints or by using synthetic chimeric antigen receptors (CARs) in T cells (CAR-T) to target tumor antigens. However, off-target immunotoxicity, inadequate solid-tumor invasion, and complex tumor heterogeneity pose significant issues in cancer immunotherapy. Modifying the physicochemical properties of nanomaterials has demonstrated considerable potential to address these issues as it allows accurate immunomodulatory delivery, controlled drug release, and improved barrier permeability. For example, micelles, liposomes, polymersomes, and other inorganic nanomaterials are widely used for delivering adjuvants, antibodies, antigens, and cytokines (Gong et al., 2019). Immunomodulators based on NPs have been developed to treat leukemia and circulating tumors; however, this strategy has encountered issues, including rapid clearance by the reticuloendothelial system. Negatively charged NPs can effectively deliver various substances by enhancing circulation half-life and facilitating cellular uptake. Conversely, the extracellular matrix rejects immune cells and NPs in the treatment of solid tumors. In this regard, small, surface-modified NPs may enable deep tissue penetration. Furthermore, polycationic NPs may facilitate endosomal escape *via* mechanical stress in such situations. Crucially, to overcome the multi-stage biological obstacles, it is necessary to investigate rational alterations to the properties of NPs (Ni Q. et al., 2022).

#### 3.1.1 NPs targeting tumor-associated macrophages (TAMs)

TAMs are crucial for controlling tumor progression. There are two different types of macrophages: M1 polarized macrophages stimulate inflammation, and M2 macrophages suppress the immune system. For example, cytotoxic T lymphocytes that help the tumor survive are suppressed by M2 macrophages. Therefore, macrophage engineering to control the tumor immune microenvironment for cancer treatment is an integral part of current immunological research. M2-TAMs may be responsible for non-Hodgkin’s lymphoma by exhibiting immunosuppressive activities (Singh et al., 2017; Reichel et al., 2019).

**FIGURE 2:**
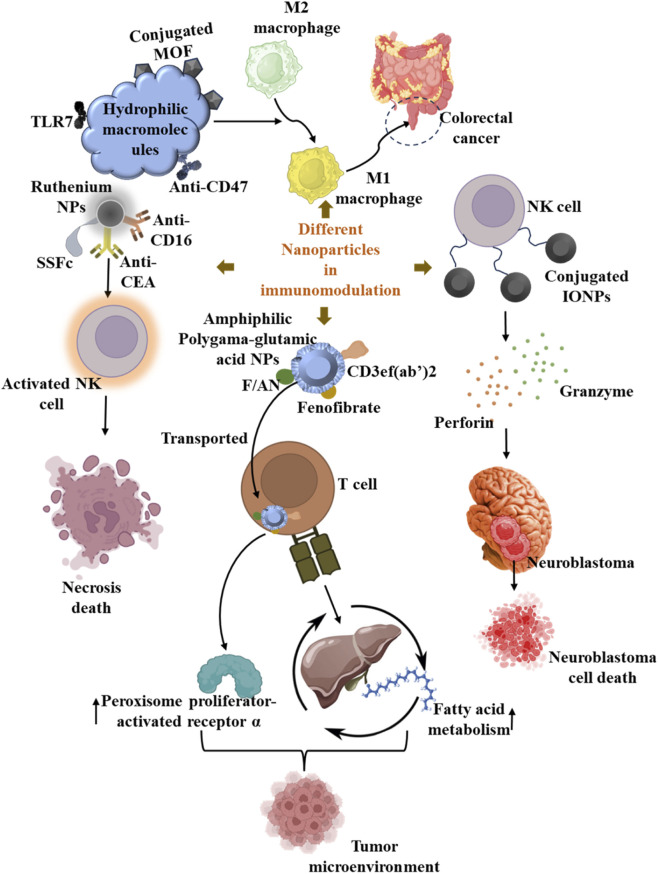
Hydrophilic macromolecular anti-CD47 antibody and a hydrophobic small-molecule TLR7 agonist conjugated to a metal-organic framework to repolarize M1 macrophages in colorectal tumor treatment. Iron oxide NPs conjugated with NK cells in neuroblastoma cells. Amphiphilic polygama-glutamic acid-based NPs (F/ANs), encapsulated within fenofibrate and surface-modified with an anti-CD3e f (ab*'*)2 fragment (aCD3/F/ANs) to activate effector T cells for anti-tumor efficacy. Ruthenium NPs modified with anti-CD16, SSFc, and anti-CEA to activate NK cells for cancer cell death.

Additionally, TAMs overexpress TGF-β and IL-10, which suppress tumor immunity and alter T cell-mediated responses (Vinogradov et al., 2014). CSF1 is another secretory molecule produced by tumor cells, which promotes TAM invasion and releases VEGF. Together, these actions allow vascularization and stimulate tumor growth. TAM-secreted TNF-α can cause tumor cell motility, and TAM-secreted IL-6 and CCL4 are associated with promoting metastasis (Singh et al., 2017). Remarkably, monocytes migrate from the bone marrow to the site of inflammation and reduce CCL2/CCR2 production, further reducing the concentration of TAMs (Lim et al., 2016). Reprogramming TAM (M2 macrophages) into an M1-polarized state is a top priority for toll-like receptors (TLRs), such as TLR3, 7, 8, and 9. In this regard, a recent study has developed a nanocarrier delivery system, in which a hydrophilic macromolecular anti-CD47 antibody and a hydrophobic small-molecule TLR7 agonist have been conjugated to a nanoscale metal-organic framework to repolarize M1 macrophages and aid in the destruction of metastatic tumors in bilateral colorectal tumor models (Figure 2) (Ni K. et al., 2022; Zhao C. et al., 2022).

#### 3.1.2 NPs in activating natural killer (NK) cells

NK cells are essential for first-line resistance towards infectious pathogens and malignant conditions. In cancer, NK cell-based therapy is becoming a viable substitute for T cell-based therapy. In this regard, nanotechnology can be used to enhance NK cells’ cytotoxicity. NPs can activate various immune cells, including NK cells, and deliver bioactive substances and medications. NK cells have been successfully activated using selenium-based NPs (Gao et al., 2020). NK cells induce cell-mediated and nonspecific humoral immune responses. Additionally, NK cells induced the expression of cytokines IFN-γ and IL-17 (Yazdi et al., 2013; Gao et al., 2020; Ferro et al., 2021). Ruthenium NPs decorated with anti-CD16, SSFc, and anti-CEA antibodies have been used to activate NK cells and induce necrosis and cell death (Figure 2) (Xu et al., 2019; Chen et al., 2022). Iron oxide NPs conjugated to NK cells have been shown to increase granzyme and perforin production in neuroblastoma cells (Figure 2) (Cifaldi et al., 2019; Murugan et al., 2022).

#### 3.1.3 Nanomaterials in T cell activation

Cancer vaccine-based monotherapy has a lower success rate than the other forms of cancer immunotherapy. Nanotechnology may improve the effectiveness of cancer vaccines by targeting the “cold” tumor immune microenvironment. A cationic polymeric NP-based formulation using PLGA and polyamidoamine dendrimers has been used as an adjuvant with tumor-associated antigen as a cancer vaccine. This formulation has increased the number of tumor-associated antigen-specific T cells in the peripheral blood and reduced tumor size in mice. This cationic polymer-based nanoformulation probably creates an inflammatory environment that further activates cytotoxic cells against cancer (Smith et al., 2022).

Another study has formulated amphiphilic polyglutamic acid-based NPs (F/ANs), encapsulated within fenofibrate and surface-modified with an anti-CD3e f (ab*'*)2 fragment (aCD3/F/ANs) to stimulate effector T cells for anti-tumor efficacy (Figure 2). aCD3/F/ANs were transported to T cells more effectively and successfully than simple and functionalized F/ANs. Additionally, upon treatment completion, T cells showed elevated levels of downstream fatty acid metabolism and genes associated with peroxisome proliferator-activated receptor-α, which facilitated their proliferation in a glucose-deficient TME. This study has also reported improved eradication of B16F10 melanoma cells against B16F10 melanoma cells and increased T cell infiltration into the TME *in vivo*. Therefore, activating T cells with nanoformulations may modulate lipid metabolism in cancer immuno-metabolic therapy (Kim et al., 2021). However, tailored delivery focused on T cell activation faces significant challenges. Antibody-targeted NPs that may bind to cytotoxic T cells in mouse tumors, lymphoid tissue, and blood are now being studied to address such challenges. Administering a TGF-β inhibitor to cells expressing PD-1 increases the survival rate of tumor-bearing animals. By targeting PD-1 in the TME, this T cell-targeted nanodelivery method also delivers a TLR7/8 agonist. To eliminate cancer cells, this system also increases the percentage of cytotoxic cells (Schmid et al., 2017).

### 3.2 Cancer cell membrane-wrapped NPs in enhancing tumor immunity

Bionic nanotechnology has emerged as a promising field owing to the potential of nanomedicine for cancer therapy. Nonetheless, the combination of immunotherapy and nanotechnology is equally crucial in boosting anti-tumor activities. Cancer cell membrane -wrapped NPs (CCNPs) are an intriguing development in nano-immuno-cancer therapy. Because several molecules, such as integrins, lymphocyte-homing receptors, mucoprotein-1, and epithelial adhesion molecules, cover the membrane of melanoma cells, CCNPs may evade the immune system (Nath and Mukherjee, 2014; Gordon-Alonso et al., 2017; Bose et al., 2018; Sökeland and Schumacher, 2019). MDA-MB-231-PLGA NPs can prevent cancer cells from migrating to fibroblast cells, and increase the number of cytotoxic T cells in immunocompetent mice (Jin et al., 2019). Fe_3_O_4_@SiO_2_ NPs coated with tumor-specific antigens stimulate NK cells to increase surface-activating receptors and boost anti-cancer activity (Wu et al., 2021). Additionally, a biomimetic nanoplatform AM@DLMSN@CuS/R848 has been developed to target TNBC. R848 adjuvant and a PD-1/PD-L1 inhibitor have been incorporated into this nano-platform, and photothermal therapy has been used to release R848, which acts as a vaccine against triple-negative cancer cells (Cheng et al., 2020).

A recent study has synthesized silica-core/gold-shell nanoshells to increase their accumulation in TNBC cells. TNBC is among the most aggressive malignancies owing to the lack of efficient medication. In comparison to EpH4-Ev non-cancerous cells, cancer cell membrane-wrapped NPs have demonstrated targeted specificity toward 4T1 TNBC with improved intratumoral apoptosis and photothermal therapy-based tumor growth inhibition (Aboeleneen et al., 2025). Therefore, the treatment strategy for many solid tumors and triple-negative breast cancer may be improved using cell membrane-wrapped NPs.

*Fusobacterium nucleatum* may colonize tumor tissues in breast cancer, which leads to immunosuppression and accelerates tumor growth. Therefore, a treatment approach targeting intratumoral bacteria is equally crucial. To explore this, a new FMV@PCFPC nanodelivery system has been developed that may induce immunogenic cell death *via* dual ferroptosis in both intratumoral bacteria and the tumor. mPEG-β-CD/α-CD (a hemirotaxane host-guest complex) and Fc-PEI (polyethyleneimine-ferrocene) have been used to generate PCFP, which was subsequently loaded with the antibacterial agent cinnamaldehyde (PCFPC). Finally, vesicles secreted by *F. nucleatum* (FMVs) were wrapped around PCFPC. FMVs aid in the absorption of cinnamaldehyde by tumor cells, and PCFPC targets cancer cells and produces ferrocene. Antigen presentation and dendritic cell maturation were improved by PCFPC-mediated tumor ferroptosis, and cancer cells were eliminated by activating cytotoxic T cells. Crucially, bacterial pieces and PAMPs on FMVs act as adjuvants to boost immune responses (Shen et al., 2025). Similarly, the intratumoral bacterium *F. nucleatum* membrane-wrapped covalent organic frameworks (COF) have been uniformly taken up by tumor tissues. In this study, the *F. nucleatum* membrane acted as an adjuvant, changing the “cold” tumor immune microenvironment into a “warm” one by increasing infiltration of B and cytotoxic cells. Therefore, this novel COF may enhance immunotherapeutic potential by integrating nanotechnology and microbial technology (Yang et al., 2025).

## 4 Two-dimensional nanomaterials in photothermal-cancer immunotherapy

Recently, photothermal therapy (PTT) has been considered as a promising method for cancer treatment (Duan et al., 2023). A photothermal agent harvests energy from near-infrared (NIR) light and converts it into heat that may raise the temperature near tumor tissues and kill cancer cells (Yang et al., 2016; Liu et al., 2018; Nam et al., 2018). Transition metals and small organic molecules are the most commonly used photothermal agents. Photothermal therapy offers excellent eradication capabilities for solid tumors, low side effects, and high specificity (Ban et al., 2017; Zhang et al., 2021; Kong and Chen, 2022). However, PTT alone has several limitations when applied to deep tumors. Notably, immunotherapy and PTT show enhanced tumor-eradicating potential and improve immunological memory (Xie et al., 2025).

### 4.1 Mechanism of photothermal therapy (PTT)

PTT involves three major mechanisms: physical mechanisms, immunological states, and activation of apoptotic pathways.

**FIGURE 3:**
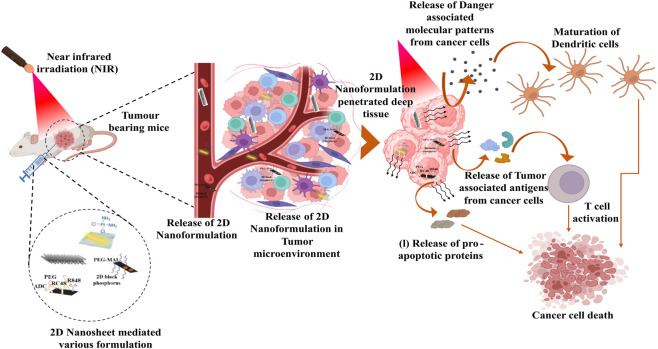
Mechanistic insights of PTT in activating immune cells to trigger cancer cell death.

In terms of physical mechanism, NIR radiation can effectively penetrate biological tissues internally and produce heat inside tumor cells; in this situation, the selection of laser wavelength is crucial. Some laser energy is lost in the NIR window after deeply penetrating tissues, and the remaining energy density is relatively high (Wu and Butt, 2016). Once photothermal transduction agents reach (Figure 3) the tissue, they transform light energy into heat in the presence of NIR radiation. This causes localized hyperthermia, which leads to necrosis or apoptosis of cancer cells. Photothermal transduction agents excited by NIR radiation reach a singlet excited state before transitioning back to the ground state in the same solid collision. This process transforms light energy into kinetic energy and generates heat around the tissue, which can be exploited to treat cancer (Figure 3) (Alkilany et al., 2012; Sheng et al., 2017). Photothermal immunological mechanisms have also drawn considerable attention. Vascular malformation renders tumor tissues to tolerate heat relatively easily, which results in immunogenic cell death (Figure 3). Tumor-associated antigens and danger-associated molecular patterns (Figure 3) mediate immunogenic death of cancer cells (Li et al., 2021). Crucially, the ubiquitous molecular chaperone heat shock protein (HSP) 70 promotes protein folding and induces anti-apoptotic effects by suppressing the production of caspase 3 at high temperatures (Ali et al., 2016). PTT-induced inhibition of HSP expression impairs the anti-apoptotic potential of cancer cells (Wang et al., 2018; Guo et al., 2020).

### 4.2 Advanced 2D nanomaterials in cancer immunotherapy

Radiation therapy plays a significant part in treating cancer. However, its use is limited owing to low DNA-damaging effects in cancer cells and potential toxicity toward non-cancerous cells. A recent study has investigated the use of 2D MXene (Table 2) as a core carrier system, coated with cisplatin and gold nanorods, to address this issue. Additionally, this entire system, known as APMP, has been encapsulated within polyvinyl alcohol. APMP produced 2.6 times more ROS than radiation therapy alone. Consequently, the combination of radiotherapy, APMP, and phototherapy demonstrated the ability to eradicate radioresistant cancer cells, highlighting this as a promising approach. Furthermore, in glioblastoma treatment, this developed system functions as a radiosensitizer, inducing immunogenic cell death (Zhu et al., 2025a). Therapeutic resistance is a big challenge in treating triple-negative breast cancer, which is extremely aggressive. This issue has been investigated by designing Ti_3_C_2_-MXene QDs with two-photon absorption coefficients based on an NIR-II probe. Additionally, Ti_3_C_2_-MXene QDs conjugated with an anti-AX antibody show higher photon absorption coefficients than do other nanomaterials, including Nb_2_C MXene QDs, MoS_2_ QDs, and black phosphorus QDs. Antibody-coated Ti_3_C_2_-MXene QDs have been able to significantly distinguish triple-negative breast cancer cells, as observed through NIR-II response live imaging with nontargeted HER-2(+), SK-BR-3 breast cancer cells, and ER+ MCF-7 breast cancer cells (Rai et al., 2026). Macrophage polarization possesses significant challenge in using nanomaterials in cancer immunotherapy. Therefore, transition metal carbide (MXene) nanosheets have been used, owing to their high electrical conductivity and magnetoelectric activity, to be mechanistically endocytosed and produce ROS. In addition to inhibiting the NF-κB and JAK–STAT (Janus kinase/signal transducers and activators of transcription) signaling pathways, ROS may help polarize macrophages under a rotating magnetic field. Therefore, 2D MXene may be useful for cancer immunotherapy (Huang et al., 2026).

**TABLE 2:** 2D nanomaterials in photothermal immunotherapy for cancer.

2D nanomaterials	Targeted cancer	Photothermal conversion efficiency (η) and NIR	Temperature	*In-vitro*/*In-vivo* model	Therapeutic effect	References
Au-Pt@Ti_3_C_2_-Mxene@PVA nanocomposites (APMP)	Glioblastoma	750–900 nm (NIR)	29.8 °C enhanced	GL261 cells/C57BL/6 mice	• Impede DNA damage repair mechanisms • Elevated reactive oxygen species (ROS) production • TNF-α and IFN-γ increased expression • CD4 ^+^ T and CD8 ^+^ T cells infiltration enhanced and decreased T reg cells population in tumor • Drive immunogenic cell death	Zhu et al. (2025a)
2D genesheet (composed with metallic-MoS_2_ and siRNA)	Breast cancer	η = 56% NIR-II (1,000–1,350 nm)	Nearly 60 °C	BALB/c mice bearing 4T1 tumors	• Attenuated IDO1 protein expression • Reverse IDO1-associated immunosuppression • Promote dendritic cell maturation with increased T cell infiltration	Zhu et al. (2025b)
FMPN (Combined with Mn-DOTA and dimeric PD-L1 affibody)	Breast cancer	η = 40.33% 808 nm NIR	52.7 °C	4T1 cells/Female BALB/c mice	• pH based Fe^2+^ release induced tumor cell ferroptosis • Enhanced PD-L1 affibody tumor targeting • Termination of T cells’ immune suppression	Zhang et al. (2025)
BP-PEG-MAL@antigen nanovaccines	Breast cancer	η = 30.84% 808 nm NIR	50 °C	BALB/C female mice bearing 4T1 cells	• Activation of dendritic cells through tumor-derived protein antigens • Dendritic cells secreted TNF-α and IL-6 to promote apoptosis, B cell proliferation, and T cell activation	Liang et al. (2025)
BP-PEG-RC48-R848 nano composites	Breast cancer	η = 30.3% 808 nm NIR	55 °C	4T1 cells/female Balb/c mice	• Dendritic cells maturation • Enhanced secretion of pro-inflammatory M1 cytokines • Effectively ablated primary local tumors	Xiao et al. (2025)

The cGAS-STING pathway has received increasing attention in relation to cancer immunotherapy. Combining cGAS-STING agonists with chemotherapeutic drugs may improve therapeutic efficacy and provide an effective cancer immunotherapeutic component. MnCl_2_ 2D nanosheets have been used in this context without the need for other drugs. Mechanistically, nanosheets cause a Mn^2+^ storm to enter tumor cells, which in turn causes cytotoxicity and DNA damage. They also induce the release of antigens and molecular patterns associated to specific tumors, which activate both innate and adaptive immune responses (Ma et al., 2026). Dynamic nanopatch platforms that respond to light and further engage in immunoactivation have been used for cancer immunotherapy. Crystalline poly (ε-caprolactone) has been used to create this platform, which has been combined with 2D photothermal conversion components. NIR light alters the morphology of this nanopatch from a 2D planar shape to a zero-dimensional spherical form. Following morphological alterations, spherical nanopatch interacts with the cell membrane through internalization, deformation, and membrane adherence. The whole process generates mechanical stress that facilitates the release of tumor-associated antigens by disrupting the membrane, which induces immunogenic cell killing (Wu et al., 2026).

**FIGURE 4:**
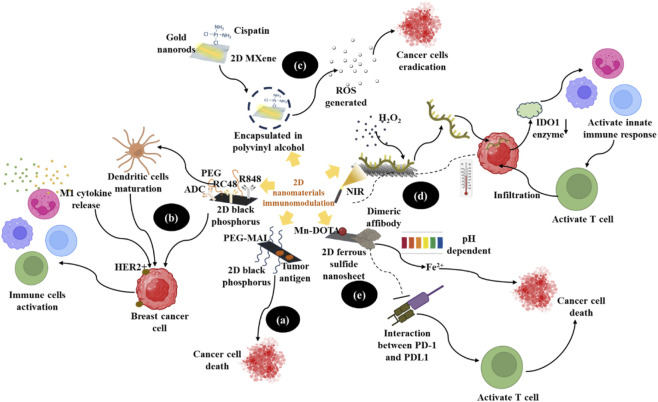
Detailed mechanism of 2D nanomaterials used in immune cell activation by **(a, b)** 2D black phosphorus **(c)** gold nanorods conjugated 2D MXene with polyvinyl alcohol **(d)** supramolecular self-assembly based on MoS_2_ and siRNA to create a 2D gene sheet **(e)** Mn-DOTA conjugated with a dimeric PD-L1 affibody on ferrous sulfide nanosheets against cancer cells by using photothermal therapy.

Another study has used a versatile supramolecular self-assembly based on MoS_2_ and siRNA to create a 2D gene sheet (Figure 4D; Table 2). This entire system has demonstrated H_2_O_2_-stimulated biodegradability and 56% photothermal conversion efficacy under NIR-II illumination. Additionally, a mechanistic investigation has revealed that siRNA administration to cancer cells activated innate immune response and reduced the expression of indoleamine 2,3-dioxygenase 1 (IDO1), an enzyme responsible for immunosuppression. In addition to helping develop dendritic cells and upsurge T cell infiltration, NIR-II-mediated phototherapy may induce immunogenic cell death (Zhu et al., 2025a).

A recent study has developed an ultrathin 2D ferrous sulfide nanosheet coupled with Mn-DOTA and a dimeric PD-L1 affibody (Figure 4e) for tumor therapy and dual-mode magnetic resonance imaging (Table 2). This modality demonstrated considerable biodegradability and a photothermal conversion efficacy of 40.33%. Consequently, it released Fe^2+^ in a pH-dependent manner (Figure 4e), causing immune cell death, in addition to exhibiting vigorous photothermal therapeutic activity against cancer cells. Additionally, by hindering the interaction between PD-1 and PD-L1, PD-L1 affibody helped improve immunological responses mediated by T cells (Zhang et al., 2025). In another study, 2D black phosphorus has been used as a nanoplatform to functionalize it with maleimide poly (ethylene glycol) (PEG) and coat it with a tumor-associated antigen protein to create a BP-PEGMAL@antigen system (Figure 4a; Table 2). The functionalization of this developed system with PEG and tumor antigen contributed to its considerable stability and biocompatibility. Additionally, it demonstrated a substantial photothermal effect on cancer cells with robust immune responses (Liang et al., 2025). Another study has focused on improving photothermal therapy and anti-cancer immunity by using black phosphorus conjugated with a tumor-selective antibody-drug conjugate, distamycin vedotin (RC48), and resiquimod (R848, a TLR7/8 agonist) in HER2-positive breast cancer (Table 2). This system, known as BP-PEG-RC48-R848 (Figure 4b), is a nanocomposite with excellent photothermal stability, which can aid in dendritic cell maturation and stimulate the release of proinflammatory M1 cytokines (Figure 4b). This nanocomposite efficiently ablated primary tumors and increased immune cell activation *in vivo* when near-infrared irradiation was applied (Xiao et al., 2025).

To support the combination of photothermal therapy and cancer immunotherapy, layered double hydroxide (LDH) 2D nanosheets have also been used. The ability of high entropy-based LDH to produce large amount of ROS to modify the TME and cause pyroptosis has been investigated. The multienzyme catalytic activity of this high-entropy LDH is improved under ultrasound irradiation. It may provide an intermediate H_2_O_2_ adsorption strength and lower the energy barrier for peroxidase-like activity. Nevertheless, to induce immunogenic cell death, pyroptosis also triggers adaptive immune response (Yang X. et al., 2026). Paraptosis plays a crucial role in cell death, in which cytoplasmic vacuolization may result as an alternative to overcoming apoptosis resistance. Nickel-doped ZnMo-LDH 2D nanosheets (DR-Ni-ZnMo-LDH) with a high capability of generating singlet oxygen can cause apoptosis, ferroptosis, and paraptosis in cancer cells that are resistant to apoptosis (Yang Y. et al., 2026). The use of NIR therapy is essential in treating deep-tissue cancer. For NIR-II mediated immunotherapy, a dual optimized photosensitizer has been developed conjugating I-functionalized isophthalic acid into ZnAl-LDH interlayers. The NIR-II excitation bandgap becomes narrow, and its triplet lifespan is extended owing to the presence of LDH. Increased apoptosis and immunogenic cell death have been noted following PEG modification of this whole system. The TME exhibited dendritic cell maturation, macrophage polarization, and cytotoxic T cell activation (Hu et al., 2026).

## 5 Critical comparison between various nanoparticles as advancements in cancer therapy

Out of the three main sections of this review, Section 2 illustrates targeting of oncogenic signaling pathways by nanoparticles, and Section 3 highlights basic possibilities of complementary approaches in developing cancer nanomedicine, focusing on immunomodulation. Interestingly, Section 2 focuses on the modulation of dysregulatory intracellular signaling pathways mediated by nanomaterials, which are directly associated with tumor growth and treatment resistance. In this context, nanoparticles enable high intracellular accumulation of anti-cancer drugs with reduced systemic toxicity, which suppresses tumor cell proliferation. However, long-term tumor-controlling potential is further limited by tumor heterogeneity and pathway redundancy, which significantly impede such therapeutic approaches. Section 3 presents an immune-centric nanoparticle-based strategy to modulate the TME by enhancing antigen presentation through membrane-wrapped nanoplatforms, increasing the cytotoxic effects of T lymphocytes, activating NK cells, and altering the function of tumor-associated macrophages. Systemic anti-cancer immunity improves immunological memory over time as a result of these nanotechnological breakthroughs. With strong tumor-specific target-based activity, these strategies, particularly bacterial membrane-wrapped nanocarriers and cancer cell membrane-wrapped nanomaterials, overcome immunological evasion and biological obstacles. Therefore, signaling pathway-targeted nanoparticles provide potent cancer cell cytotoxicity; however, immunotherapeutic nanoparticles provide long-term immunological potential.

Section 4 offers several nanotechnological breakthroughs, including the introduction of 2D nanomaterials for precise cancer treatment and immunomodulation. In the presence of NIR irradiation, 2D nanomaterials are relatively highly applicable owing to their photothermal activity, which produces heat inside tumor tissue to trigger apoptosis and immunogenic cell death. In contrast to traditional drug delivery based on nanoparticles, photothermal transduction agents offer real-time external adjustment of therapeutic intensity and overcome dose-limiting toxicity. However, the release of tumor-associated antigens and damage-associated molecular patterns that trigger antitumor activity is also facilitated by PTT-mediated immunogenic cell death. PTT based on single 2D nanomaterials mechanistically links immunotherapy and cytotoxicity, which independently occur owing to immunomodulatory action and cell signaling pathway-targeted nanoparticles. Crucially, 2D nanomaterials provide a multi-mechanism-based therapeutic effect, which is a significant improvement over previous nanoparticle-based approaches that entail immune regulation and pathway suppression.

## 6 Current nano-therapeutic formulations for clinical testing in cancer treatment

The use of nanoformulated medications has yielded promising preclinical results in cancer treatment, and these are now advancing toward clinical trials. For example, polylysine-protected double-stranded RNA, also known as poly-ICLC, which functions as a TLR3 agonist, has shown promising clinical prospects (Caskey et al., 2011). Subcutaneous administration of poly-ICLC (Table 3) triggered both inflammasome signaling and an IFN-1-type immune response. Following conjugation to peptide antigens or incorporation with an immune checkpoint inhibitor, poly-ICLC demonstrated strong adjuvanticity and dendritic cell targeting in phase I/II clinical trials for TNBC, colon, glioblastoma, and ovarian cancer. Monophosphoryl lipid A (MPL) has been combined with CpG 7909 and QS-21 to formulate AS15. AS15 (Table 3), combined with MAGE-A3 (melanoma antigen family A, 3), triggered a T cell-facilitated immune response and the production of MAGE-A3-specific antibodies in a phase II clinical trial involving patients with melanoma (Kruit et al., 2013). Presently, it is in phase III clinical trials for non-small-cell lung cancer (NCT00480025) and surgically resected melanoma (NCT00796445). Another clinical trial using the same formulation has been investigated in patients with bladder cancer (NCT01498172), in which the MAGE-A3+AS15 vaccine was modified with *Bacillus* Calmette-Guerin in a phase I study.

**TABLE 3:** Nano-immuno delivery systems in clinical trials for different cancer immunotherapies.

Product names	Nanomaterials	Targeted immune cells	Types of cancer	Route of administration	Clinical identifiers	References
Poly ICLC	Polylysine/carboxymethylcellulose	Dendritic cells	Multiple cancers	Intramuscular	NCT00374049/NCT02834052/NCT03721679/NCT00986609/NCT02166905/NCT02078648	Caskey et al. (2011), Yu et al. (2022)
AS15	Liposome	Antigen-presenting cells	Multiple cancers	Intramuscular	NCT00952692/NCT01498172/NCT02364492/NCT01435356/NCT00148928/NCT01853878/NCT00058526/NCT00086866/NCT00796445/NCT00480025/NCT01266603/NCT00140738/NCT01159964/NCT01149343/NCT01220128	Kruit et al. (2013), Yu et al. (2022)
L-BLP25	Liposome	dLN-Antigen-presenting cells	Multiple cancers	Subcutaneous	NCT01423760/NCT01462513/NCT01094548/NCT01496131/NCT01731587/NCT01507103/NCT00157196/NCT00960115/NCT01015443/NCT00157209/NCT00828009/NCT00409188/NCT00925548/NCT02049151	Palmer et al. (2001), Butts et al. (2011), Yu et al. (2022)
DPX-Survivac	Liposome (DPX)	Antigen-presenting cells	Multiple cancers	Subcutaneous	NCT04895761/NCT01416038/NCT02785250/NCT03029403/NCT03332576/NCT03836352/NCT03349450/NCT05243524/NCT04920617	Berinstein et al. (2015), Yu et al. (2022)
MelQbG10	VLP	Antigen-presenting cells	Melanoma	Subcutaneous	NCT00306566/NCT00306514/NCT00306553/NCT00651703/NCT00324623	Yu et al. (2022)
ALT-803	IL-15 superagonist complex nanogel	Cytotoxic T cells and NK cells	Solid tumors or hematological malignancies	Subcutaneous/Intravenous/Intraperitoneal	NCT01727076/NCT03563157/NCT03647423/NCT03586869/NCT03387098/NCT03329248/NCT02890758/NCT02523469/NCT02559674/NCT02384954/NCT01885897/NCT03127098/NCT02099539/NCT03054909/NCT03365661/NCT01946789/NCT03050216/NCT02138734/NCT03022825	Margolin et al. (2018), Romee et al. (2018), Rosser et al. (2018), Wrangle et al. (2018), Yu et al. (2022)
BNT111	Lipoplex	pDCs, and macrophages	Melanoma stage III/IV	Intravenous	NCT04526899/NCT02410733	Yu et al. (2022)
JVRS-100	Liposome	Peripheral immune cells	Leukemia	Intravenous	NCT00860522	Yu et al. (2022)
PRECIOUS-01	PLGA	iNKT and Antigen-presenting cells	Advanced solid tumor	Intravenous	NCT04751786	Yu et al. (2022)

L-BLP25 (Table 3), a liposomal nanoformulation, has been studied as a cancer-associated mucin 1 vaccine in phases I, II, and III, and it induced T cell-mediated immune response in patients with non-small cell lung cancer (Palmer et al., 2001). Subcutaneous administration of L-BLP25 (Table 3) has demonstrated considerable survival time in a phase IIb clinical trial (Butts et al., 2011). Patients with ovarian tumors have shown a relatively high polyfunctional T cell-mediated immunological response while administering DPX-Survivac mediated vaccine formulation with surviving HLA peptides (NCT01416038) in a phase I trial (Berinstein et al., 2015). MelQbG10 (Table 3), a highly immunogenic peptide-based vaccination resembling a virus, has positively affected patients with advanced melanoma through T cell-mediated recognition of melanoma antigen. Patients with melanoma have demonstrated good tolerance and activation of the cytotoxic T cell-facilitated immune response in phase I/II clinical trials (Speiser et al., 2010). Remarkably, in a phase II clinical trial in patients with melanoma (NCT00651703) (Goldinger et al., 2012), combining adjuvants with MelQbG10 has improved the response of effector T cells with excellent memory. A recombinant IL-15 supraagonist nanogel complex called ALT-803 (Table 3), which has been tested in phase I, II, and III clinical trials for several cancer types, for example, colon, melanoma, breast, NSCLC, and renal carcinoma, has been targeted by NK and T cells. Phase I clinical trials have revealed that patients with cancer had relatively high NK cell expansion (NCT01727076) (Margolin et al., 2018), increased anti-tumor activity (NCT02523469; NCT02138734 (Rosser et al., 2018; Wrangle et al., 2018), and increased cytotoxic T cell expansion (NCT01885897) (Romee et al., 2018; Yu et al., 2022).

## 7 Translational challenges in nanomedicines

Nanomedicines applicable in clinical settings are scanty despite considerable improvements. Typical challenges in translating nanomedicines from preclinical to clinical stages include lack of novel formulations for *in vivo* effectiveness, specific regulatory compliance, and nonclassical sponsorship. Additionally, various technical factors make the clinical application of nanomedicines unfavorable. For example, nanomedicines become less stable than dosage forms owing to the production of nanoscale particles and their large surface area, which causes aggregation, change from amorphous to crystalline structure, and hygroscopicity. Certain nanomedicines need a suitable storage environment, the setting of which may be difficult for low-income nations (Muthu and Feng, 2009). The administration route is another translational challenge because many nanomedicines are administered parenterally, which is less effective than other non-invasive methods, including oral administration. When administered systemically, nanomedicines bind to plasma proteins and release their carriers early, which causes aggregation and immunogenicity (Desai, 2012). Additionally, in preclinical research, a body weight ratio between the experimental model and humans may result in a relatively low clinically significant dose for intravenous delivery of nanomedicines (Park, 2019). As a result, the accumulation ratio of nanomedicines in human tumors is incorrectly calculated, which is another translational challenge (Younis et al., 2022).

### 7.1 Safety

Oral administration is the most convenient and safe method for nanomedicines. However, poor stability, low bioavailability, and poor solubility of nanomaterials are often noticed owing to high rate of enzymatic breakdown in the gastric environment. In addition, the mucus layer of the intestinal wall and tight connections between the epithelium may cause inadequate absorption (Younis et al., 2022). Another safety concern of nanomedicines is the biosafety of active pharmaceutical ingredients (APIs). Changes have been reported following the formulation of the API in nanomaterials, including effects on biodistribution and their accumulation in major organs, such as the kidney and lymphoid organs, which increase toxicity. Furthermore, nanocarriers can cause severe hypersensitivity reactions; therefore, studying their interactions with various body components is necessary during clinical transition (Fogel, 2018; Szebeni et al., 2018; Metselaar and Lammers, 2020).

### 7.2 Scalability

Microfluidic devices have shown promising results to increase the scalability of nanomedicines by decreasing production time and steps (Younis et al., 2022). Numerous variables, such as mixture composition, concentration of polymers in solution, flow rate ratio, and overall flow rate, can be adjusted in such devices (Leung et al., 2015). Ultra-small nanoparticles that can readily penetrate the blood–brain barrier and reach the TME can also be generated using microfluidic devices (Belliveau et al., 2012; Teleanu et al., 2018). Good scalability of nanomedicine can be generated in industry because of the rapid synthesis method and decreased step involvement (Maeki et al., 2018). It may also be possible to optimize many formulations for greater scalability through economical screening using quick and high-throughput techniques.

### 7.3 Regulatory hurdles

Despite several regulatory issues, nanomedicine is an emerging research field with several branches, such as drug delivery, regenerative medicine, and theranostics. The main hurdles are related to the pharmacokinetic and pharmacodynamic profiles of nanomedicines (Đorđević et al., 2022). Furthermore, a lack of global agreement exists regarding classified nanotechnology that focuses on medications and medical devices. As a result, regulatory responsibilities for the nanomedicine framework varies globally, which further complicates the approval process (Đorđević et al., 2022). Furthermore, standard guidelines for adequate characterization of nanomedicine in physicochemical and biological evaluation are absent, which complicates the entry of nanomedicines into clinical trials (Gaspar et al., 2014; Sainz et al., 2015). Therefore, the progression of nanomedicines from preclinical to clinical advancements is slow and biased. As a result, several contradictory findings have been reported (Prinz et al., 2011; Begley and Ellis, 2012).

## 8 Gaps of nanotherapeutic interventions toward a scope of nano photo-immunotherapy

Cancer-associated signaling pathways, including PI3K/Akt/mTOR, NF-κB, and MAPK pathways (Basu et al., 2009; Rahman et al., 2025; BharathwajChetty et al., 2026), were the only focus of nanotechnology-based drug delivery systems (Deb and Jain, 2024; Deb et al., 2024; 2025; Sharma et al., 2024) addressing molecular-level gaps in drug delivery research. Currently, medication delivery techniques that target cell signaling are shifting toward immunological and photothermal nanotherapeutics. Stimulating NK cells, T cells, or macrophages is the primary focus of nano-immune drug delivery systems. Researchers have frequently overlooked the need to modify intracellular tumor signaling pathways that support immune evasion mechanisms, such as PD-L1-facilitated T cell exhaustion, in favor of focusing on stimulating immune cells *via* drug delivery systems. Additionally, a novel application of nanotechnology in cancer therapy is the photo-thermal nano-immune system that has demonstrated promising results. Nonetheless, mechanisms explaining how heat-induced cellular stress affects oncogenic signaling or promotes the development of long-term immunological memory are largely unexplored. Thorough reprogramming of immunosuppressive cells using hybrid or multifunctional nanoplatforms is another significant gap. As a result, creating a multifunctional nano-immuno drug delivery system with an emphasis on photothermal therapy and cancer-related signaling pathways may be a key goal for future studies.

## 9 Conclusion and prospects

NP-based cancer treatment has emerged as a revolutionary approach in precision oncology, addressing a range of issues related to drug release profiles, target selectivity, and control of cellular signaling pathways linked to cancer. Exosomes and virus-like NPs, subtypes of biologically generated NPs, have demonstrated immunomodulatory benefits compared to those achieved by organic, inorganic, and biological NPs. Early research on NP-based cancer treatments has focused on designing NPs to enhance their anti-cancer effects. However, this focus has shifted toward targeting cancer-related signaling pathways, such as GPCR, MAPK, PI3K/Akt/mTOR, Wnt-β-catenin, and Nrf2.

Additionally, modulation of immune cell activation is another field of interest in cancer therapy. NPs have provided a novel means of delivering cytokines, antigens, and antibodies while reducing systemic toxicity. Some NPs have been designed to enhance anti-cancer immune responses, potentially by stimulating T cells, NK cells, and macrophages. Co-delivery of antibodies using a metal-organic framework has shown encouraging preclinical efficacy in reprogramming immune-suppressive M2 macrophages into an M1 phenotype. Cancer cell membrane-wrapped NPs have also emerged as a bionic technique to target immune evasion in immune-responsive delivery systems. Through photothermal therapy, this biomimetic nanoplatform provides synergistic benefits beyond T cell activation. This nano-immuno-photothermal treatment modality increases immunogenic cell death and tumor-specific apoptosis. Numerous 2D nanomaterials have demonstrated impressive results in photothermal immunotherapy, including MXene, MoS_2_, black phosphorous, graphene, and ferrous sulfide. Poly-ICLC, AS15, L-BLP25, ALT-803, and DPX-Survivac are among the nano-immunotherapeutic systems with clinical setting transition underway.

Advances in the nano-immunotherapeutic concept, integrating multifunctional hybrid systems to modulate signaling pathways in cancer, activate immune cells, and provide local photothermal effects, have become a prospect for clinical studies. Next-generation nano-photo-immunotherapy should focus on.

- Designing a biodegradable nanoplatform that may provide a pathway target, as well as reprogramming immune cells.
- Introducing AI-based nanocarrier design and optimization for precisely targeting immune cells.
- In-depth molecular mechanisms for improving immunological memory.
- Boosting the transition of the preclinical studies to translational pipelines for accelerating clinical setup, focusing on co-delivery dose management.

## Glossary

2D: Two-dimensional
NPs: Nanoparticles
EPR: Enhanced permeability and retention
VLPs: Virus-like particles
TME: Tumor microenvironment
GPCR: G-protein-coupled receptor
IONPs: Iron oxide NPs
CCK2R: GPCR cholecystokinin-2 receptor
CXCL: C-X-C motif chemokine ligand
CXCR: C-X-C motif chemokine receptor
siRNA: small-interfering RNA
PLGA: Poly(lactide-co-glycolide)
PI3K: Phosphatidylinositol 3-kinase
MAPK: Mitogen-activated protein kinase
mTOR: mechanistic target of rapamycin
KP–SeNP: *Kaempferia parvifora* extract and selenium nanoparticle
AuNTs: Gold nanotriangles
CSNP–BET: Chitosan NPs encasing betanin
TNBC: Triple-negative breast cancer
CA: Carnosic acid
TAMs: Tumor-associated macrophages
PD-L1: Programmed death-ligand 1
TNF-α: Tumor necrosis factor alpha
IL: Interleukin
NF-κB: Nuclear factor kappa B
ROS: Reactive oxygen species
Keap1: Kelch-like ECH-associated protein 1
CARs: Chimeric antigen receptors
TLRs: Toll-like receptors
F/ANs: Formulated amphiphilic polyglutamic acid-based NPs
CCNPs: Cancer cell membrane -wrapped NPs
COF: Covalent organic frameworks
PTT: Photothermal therapy
IDO1: Indoleamine 2,3-dioxygenase 1
JAK: Janus kinase
STAT: Signal transducers and activators of transcription)
